# Dataset of the palaeozoic (Carboniferous – Permian) Kinta Limestone Pinnacles in Kampar, Perak, Malaysia

**DOI:** 10.1016/j.dib.2026.113207

**Published:** 2026-08-27

**Authors:** Wei Chung Khor, Mohd Shafeea Leman, Muhammad Ashahadi Dzulkafli, Nurul `Amalina Md. Nor, Mat Niza Abdul Rahman

**Affiliations:** a Geology Programme, Faculty of Science and Natural Resources, Universiti Malaysia Sabah (UMS), 88400 Kota Kinabalu, Sabah, Borneo, Malaysia; b Geology Programme, Faculty of Science and Technology, Universiti Kebangsaan Malaysia, 43600 UKM Bangi, Selangor, Malaysia; c Department of Mineral and Geosciences Malaysia (JMG), Level 9, Menara PJH, No. 2, Jalan Tun Abdul Razak, Precinct 2, 62100 Putrajaya, Malaysia

**Keywords:** Carbonate, Sedimentology, Facies characterization

## Abstract

This dataset documents the Paleozoic Carboniferous - Permian Kinta Limestone Pinnacles in Kampar, Perak, Malaysia, preserving a geological record of a now-demolished carbonate formation. It includes sedimentary logs, facies descriptions, fossil records, petrographic images, and X-ray diffraction (XRD) analysis results, enabling in-depth studies on carbonate depositional environments, diagenetic processes, and fossil assemblages. A total of 27 pinnacles were mapped and digitized using ArcGIS 10.8, with seven correlatable pinnacles selected for detailed sedimentary logging (1:10 scale). Key attributes recorded include lithology, sedimentary structures, texture, fossil content, and diagenetic indicators, classified into five distinct facies. Hand samples were analysed using XRD to determine mineralogical composition. Given the extensive recrystallization of the limestone, thin-section observations were challenging. Fossils including fusulinids, corals, and brachiopods were documented through macro-photography for further identification. This dataset is valuable for paleoenvironmental reconstructions and comparative carbonate studies, ensuring continued access to critical geological information despite the loss of the physical site.

Specifications Table

**Table:** 

Subject	Earth & Environmental Sciences
Specific subject area	Geology; Sedimentology; Limestone, Palaeontology
Type of data	Table, Image, Filtered Image, Processed Image, Graph, Microphotograph and Map
Data collection	This data contains sedimentary logging of limestone pinnacles at Kampar, Perak, Malaysia. The several key features recorded are lithology, texture, structural data bedding orientation, faults & joints), weathering patterns, photographs, coordinates for georeferencing, sedimentary structures, diagenetic indicators and fossil content. The logging is done during field excursion with hand lens (10x), Jacob’s Staff, geological compass, grain size chart, camera, GPS and rock hammer. These data were classified into Facies (intervals) and digitized through Adobe Illustrator and exported as image (JPEG). Three hand samples were collected with geological hammer and grinded into powder form with mortar for X-Ray Diffraction (XRD) analysis using Bruker XRD D2 Phaser. XRD analysis would provide information about crystal structure which would be used to identify the mineral content.
Data source location	The field excursion site is located at Kampar District, Perak, Peninsula Malaysia. Decimal Degrees Coordinate: 4.316,789, 101.130,865
Data accessibility	Repository name: Mendeley Data Data identification number: 10.17,632/rvdcsj78j8.1 Direct URL to data: https://data.mendeley.com/datasets/rvdcsj78j8/1
Related research article	None

## 1 Value of the Data

• This data is valuable because it captures the geology of (Paleozoic) Carboniferous to Permian Kinta Limestone.
• The rock pinnacles were demolished due to urban development, making it impossible to revisit the site for further data collection.
• This data enables researchers worldwide to virtually explore the now-demolished limestone pinnacles. Essential information – including fossil content, lithology, and sedimentary structures has been meticulously documented and preserved in a digital format, ensuring continued access for scientific analysis and future studies.
• Researchers can use this dataset to reconstruct ancient carbonate depositional environments, gaining insights into sea levels during Permian – Carboniferous period, paleoclimates, and tectonic settings.
• In-depth studies of carbonate facies, diagenetic processes, dolomitization, fossil assemblages, and mineralogical compositions can be conducted, while the dataset also serves as an analogue for pre-K-T mass extinction carbonate systems worldwide.

## 2 Background

This dataset was compiled to document and preserve geological information from the Kinta Valley, specifically focusing on Permian to Carboniferous carbonate rocks. The region’s distinctive limestone pinnacles, which were once key features of the carbonate system, are no longer available for direct study due to natural and anthropogenic changes. By capturing data on these geological formations, this dataset contributes to the broader understanding of carbonate system evolution and facilitates comparative studies with other carbonate settings.

**Fig. 1:**
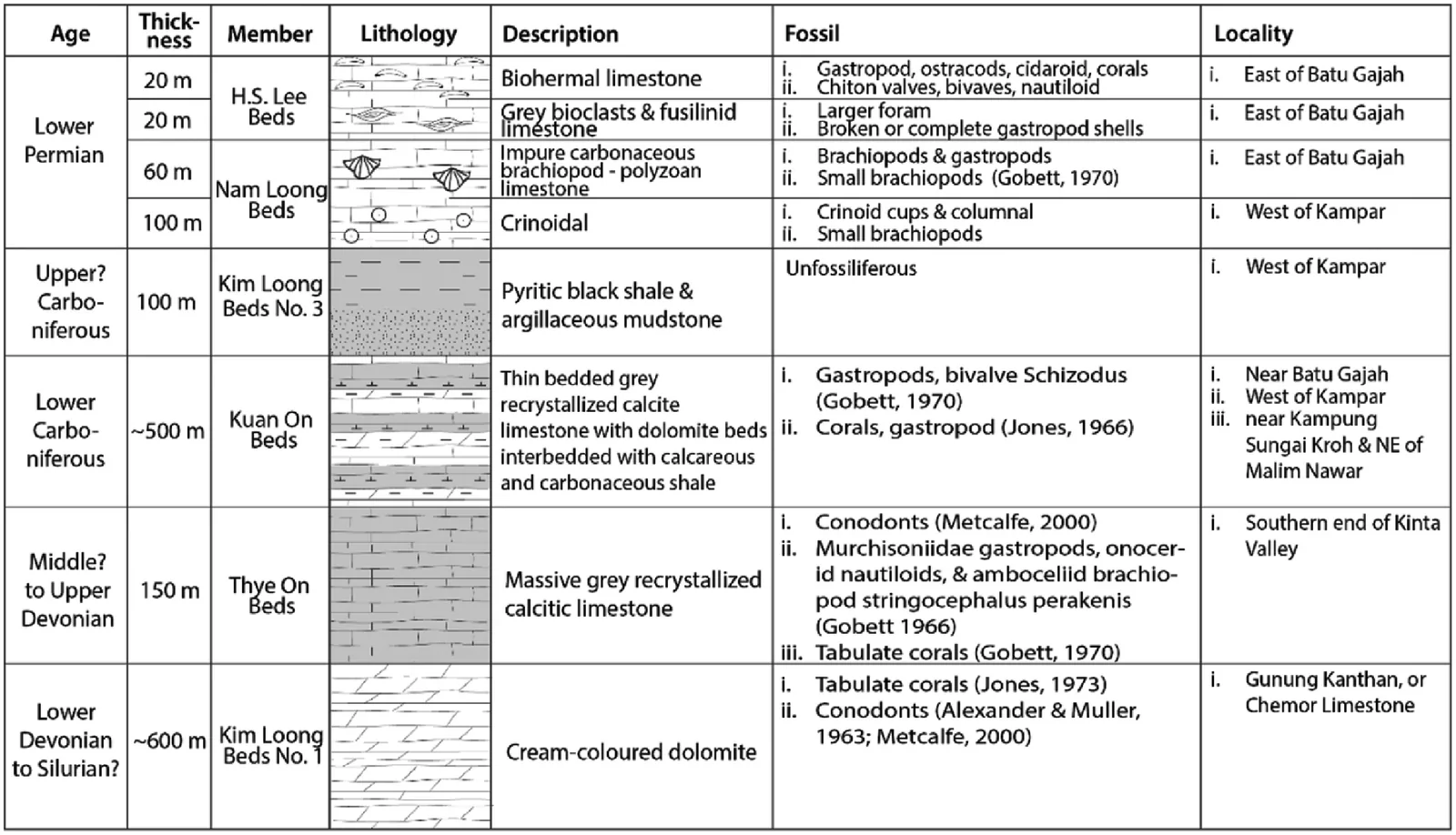
The breakdown of Kinta Formation after [1].

**Fig. 2:**
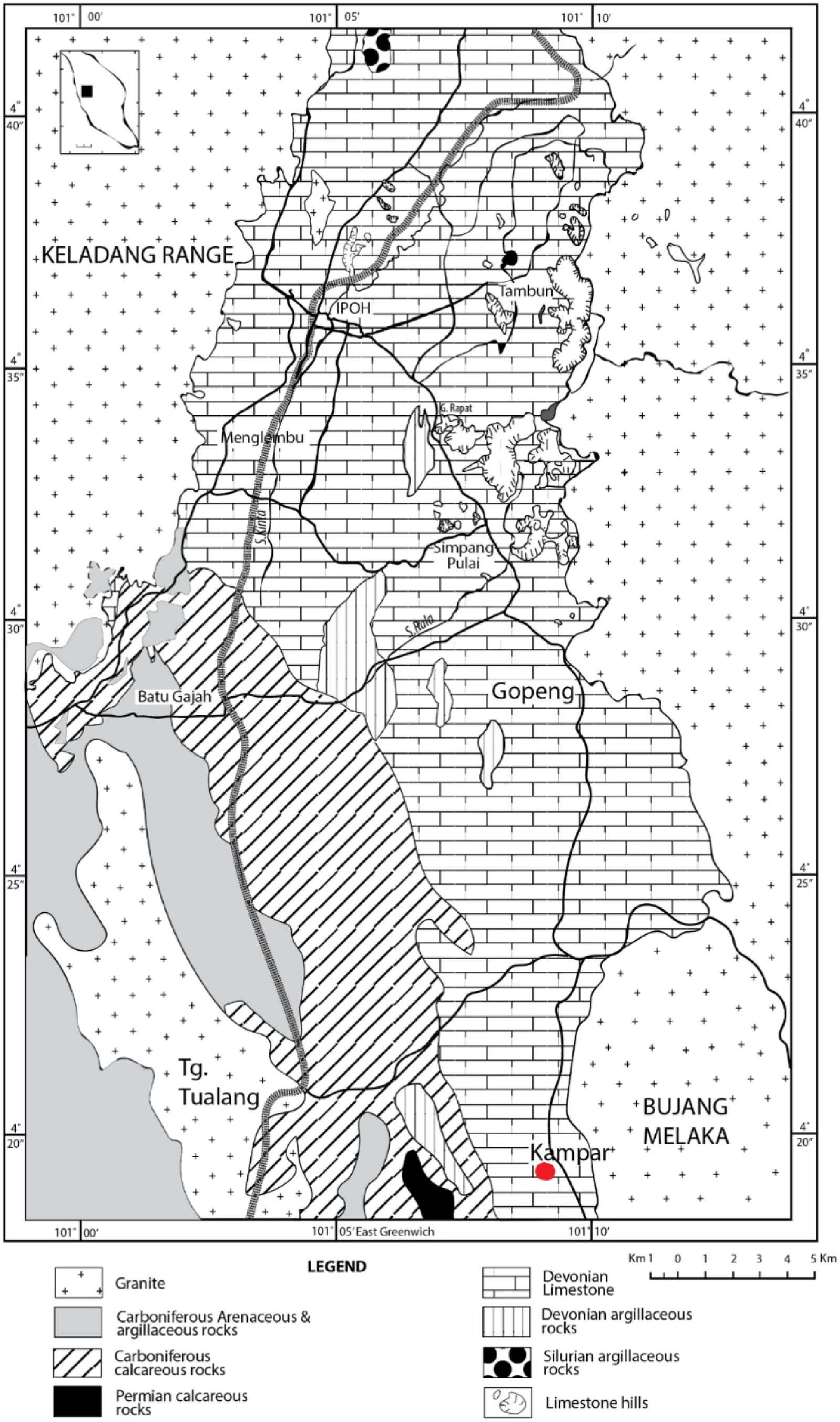
Geological map of Perak, Malaysia. The red dot indicates the location where the dataset was recorded (modified after [4]).

Kinta Formation is divided into six members, namely: i) Kim Loong Beds No.1, ii) Thye On Beds, iii) Kuan On Beds, iv) Kim Loong Beds No. 3, v) Nam Loong Beds, and vi) H.S. Lee Beds (Fig. 1). This formation is stratigraphically estimated to be 1500 m thick and spans from Batu Gajah to Chemor, Perak, Malaysia (Fig. 2). Several studies have interpreted its depositional environment as shallow marine with a wide range of settings, ranging from ramp, platform to shelfal [2,3].

## 3 Data Description

The dataset consists of 11 folders, organized as follows: (1) Maps and General Stratigraphy, (2) Sedimentary Logs, (3) – (7) facies photos, (8) Fossils Photos, (9) Pinnacle Photos, (10) X-Ray Diffraction (XRD) Analysis, and (11) Petrographic Photographs (Thin Sections).

Folder (1) contains files of geological map, locality map, and mapped pinnacles. The files are in pdf and picture format. Folder (2) includes logged continuous sections of the pinnacles. Folder (3) – (7) contain photographs of the five categorized facies. Folder (8) compiles photographs of fossils with unreferenced identifications, while folder (9) consists of pinnacle images. Information on XRD analysis is stored in folder (10). Lastly, folder 11) contains raw Plane-Polarized Light (PPL) petrographic photographs of thin sections.

## 4 Experimental Design, Materials and Methods

Data collection involves field observation which records the geological and sedimentological features through mapping and logging of the carbonate pinnacles at Kampar, Perak, Malaysia (Coordinate: 4.316,789, 101.130,865). The tools used are hand lens (10x), Jacob’s Staff, geological compass, grain size chart, camera, GPS and rock hammer.

### 4.1 Mapping

The map presented in this study was modified from [4] using Adobe Illustrator 2025. The geological mapping methodology followed the standard guidelines outlined in the official publication [5].

### 4.2 Sampling

Chip sampling method was used to collect representative rock by chipping small fragments from the outcrops. Weathered surface of the rock was first removed to obtain fresh and uncontaminated rock. The collected chip fragments were approximately 5 cm x 5 cm in size. Each sample was stored in an air-sealed sample bag and labelled. The sampling locations and geological map are provided in Folder 1 of the dataset.

### 4.3 Thin section preparation

Thin section petrography is an important technique for identifying and interpreting the textural and mineralogical characteristics of rocks. It enables detailed observation of grain relationships, crystal morphology and intergrowth, fossil components, and other microstructural features that are not readily distinguishable in hand specimens. The following procedure was adopted for the preparation of thin sections:

I. Sample cutting. Rock samples were initially cut using a rock trimmer to dimensions of approximately 4.00 cm x 1.50 cm x 0.5 cm.
II. Surface flattening. The cut surface of each rock specimen was further flattened progressively using corundum powder of 180, 320, and 600 grit. The coarser abrasives were applied using the lapping wheel, whereas the finer abrasive was applied manually on a glass plate.
III. Progressive surface grinding. The observation surface was further flattened progressively using corundum power of 180, 320, and 600 grit. The coarser abrasives were applied using the lapping wheel, whereas the finer abrasive was applied manually on a glass plate.
IV. Preparation of adhesive. Resin and hardener were mixed at a 3:2 ratio and thoroughly stirred using a glass rod for approximately 1 min. The mixture was stirred slowly to minimize the formation of air bubbles.
V. Mounting and curing. The flattened surface of the rock specimen was mounted onto the frosted surface of the glass slide using the prepared adhesive. The mounted specimen was left to cure overnight at room temperature until the adhesive had fully hardened.
VI. Final manual thinning and thickness assessment. The specimen was further thinned manually using 320 and 180 grit corundum powder. The thin section was periodically examined under a petrographic microscope to assess its thickness and optical quality.

### 4.4 Lithology

Lithology identification of these carbonate pinnacles is done in accordance with the Dunham Classification [6]. The classification classifies the rocks based on texture, clasts, mud composition, the grain framework, and the overall fabric of the rock. The classification is commonly used to distinguish carbonate rocks such as mudstone, wackestone, packstone and grainstone. The Dunham classification diagram was used to identify the lithology by observing the texture and grain-mud relationship of each limestone sample. Rocks containing >10% grains and supported by mud are classified as wackestone, while grain-supported rocks with little or no carbonate mud are classified as grainstone. The classification was applied to the logged sections. Wackestone and grainstone were among the common textures identified in the studied carbonate pinnacles.

### 4.5 Sedimentary logging

A total of 27 pinnacles were mapped and digitized using ArcGIS. Each pinnacle was systematically labeled for reference, and their position was mapped using a combination of GPS coordinates and traversing techniques to ensure spatial accuracy. From these, seven correlatable pinnacles were selected for detailed sedimentary logged. The logging process followed the methodologies outlined by Tucker and Wright (2009) [7] and Flügel (2004) [8], which provide standardized approaches for describing and interpreting carbonate and siliciclastic facies. A Jacob’s Staff, marked with distinct measurement increments, was used to obtain accurate thickness measurements of the rock layers. The logging process adhered to a detailed scale of 1:10, allowing for high-resolution documentation of vertical facies variations.

**Fig. 3:**
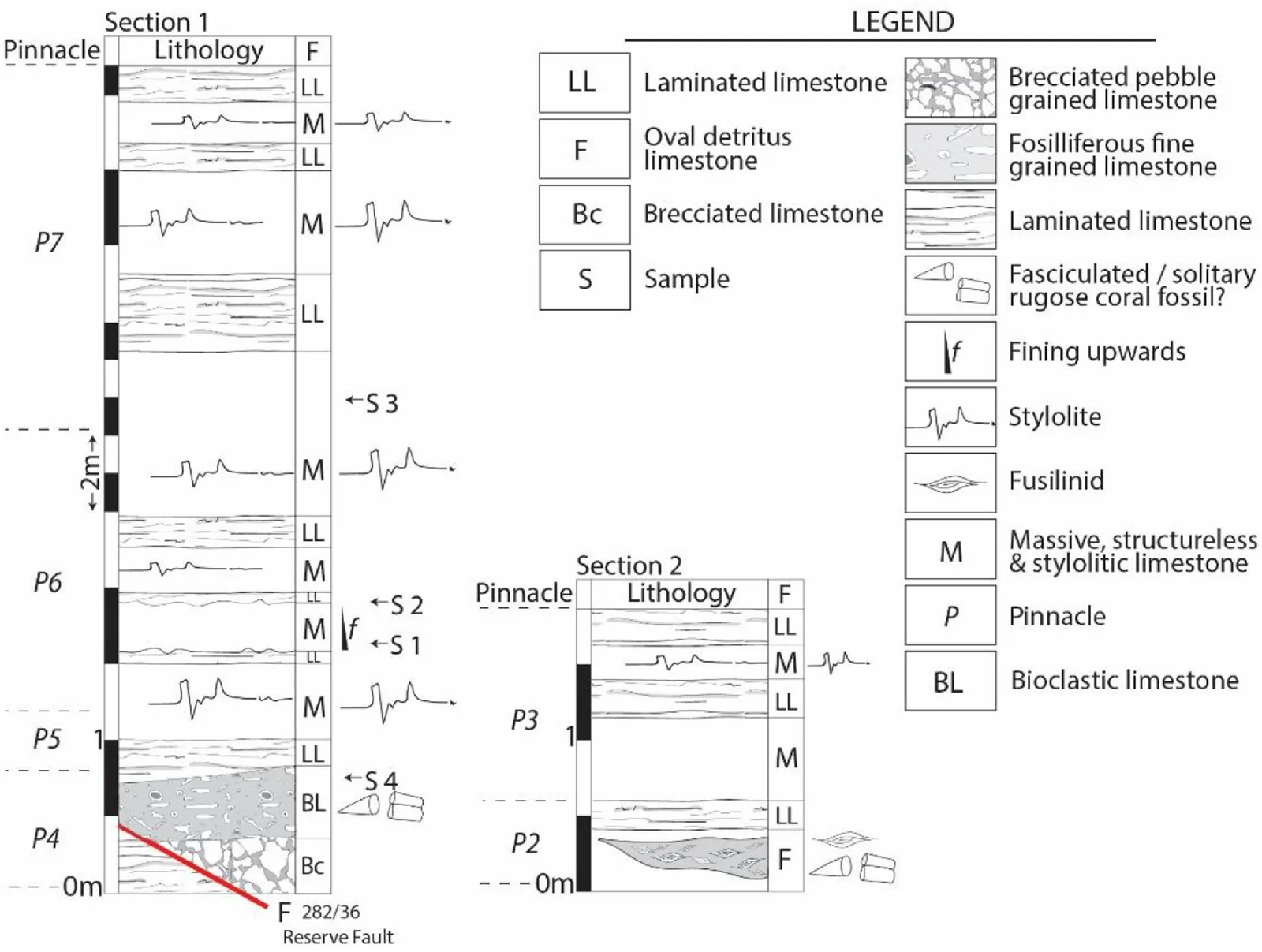
Continuous logged sections of the limestone pinnacles. Total thickness of Section 1 is 9.25 m and Section 2 is 2 m.

**Fig. 4:**
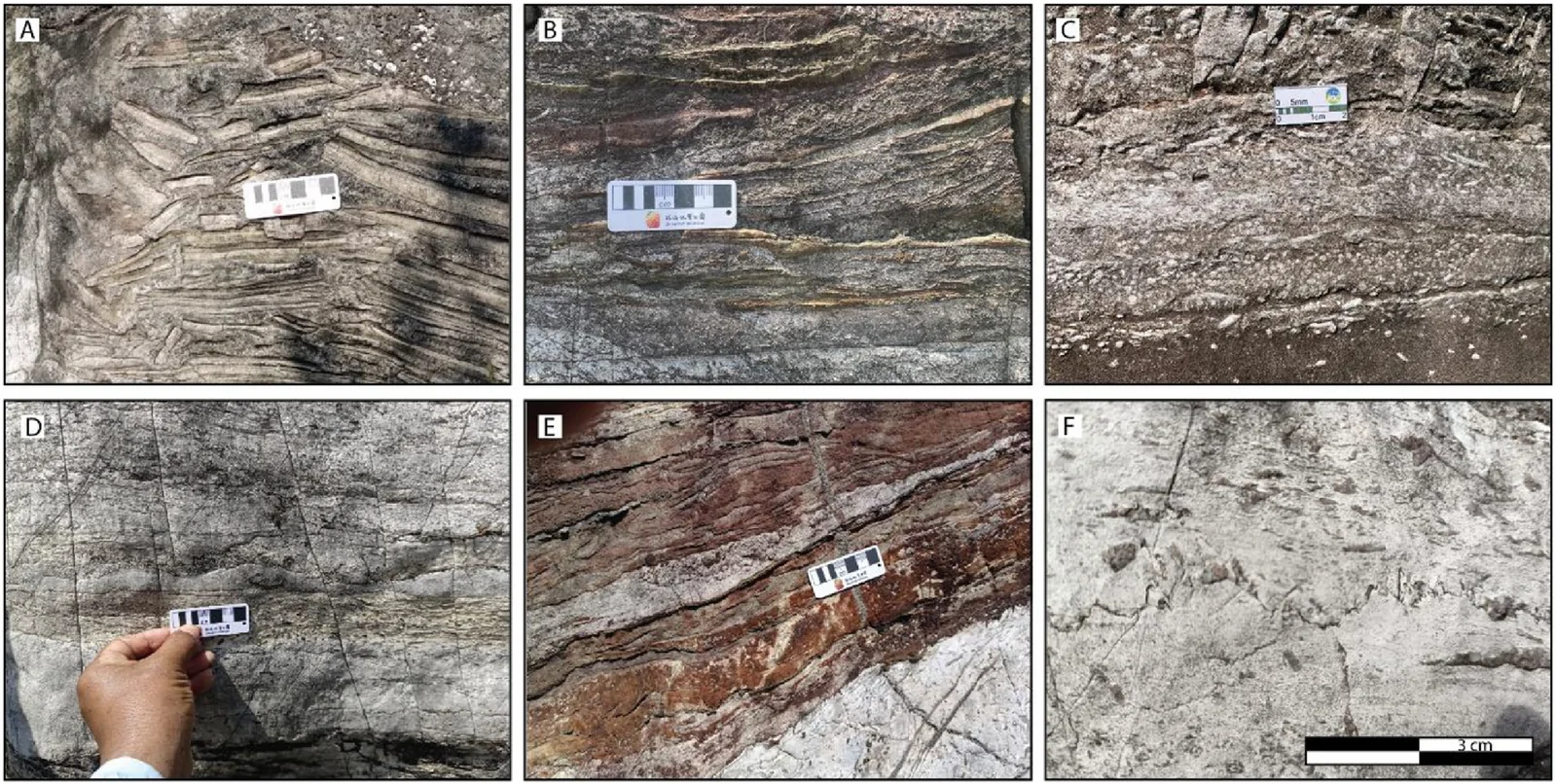
Facies identified in this dataset. A) Crystallized platy calcite in Laminated Limestone Facies (LL), B) Trough stratification in LL Facies, C) Rounded and elongated bioclasts in Bioclastic Limestone (BL), D) Structureless Limestone (M) with minor lamination, E) Laminated Limestone Facies (LL), and F) Stylolite structure in Stylolitic Limestone (M).

During the logging process, multiple sedimentological attributes were systematically recorded, including color (using Munsell color charts for consistency), sedimentary structures (e.g., lamination, crossbedding, bioturbation), grain size and texture (following the Wentworth grain size scale), and fossil content (taxonomic identification where possible). Data were recorded on standardized logging sheets, which are stored in folder (2). Following field data collection, the logs were digitized using Adobe Illustrator to enhance clarity and standardization. Then the finalized stratigraphic logs were exported as JPEG files for integration into further sedimentological analysis and interpretation (Figs. 3 and 4).

### 4.6 Facies characterization

Facies characterization was done based on the features measured (lithology, grain size, sedimentary structures, fossil content, and geochemical properties). Five distinctive facies identified are i) Stylolitic Limestone Facies (M), ii) Bioclastic Limestone Facies (BL), iii) Breccia Limestone Facies (Bc), iv) Laminated Limestone Facies (LL), and v) Oval Detritus Limestone Facies (F). The breakdown of these facies are as listed in Table 1.

**Table 1:** List of the five facies identified.

No	Facies	Code	Color	Texture	Description	Fossils	Boundaries	Thickness
1	Stylolitic Limestone Facies	M	Greyish white and minor yellowish hue	Mudstone	Structureless, stylolitic	Gastropod	Irregular and planar lower contacts	20 cm to 4 m
2	Bioclastic Limestone Facies	BL	Grey micrite and greyish white bioclasts	Wackestone and minor grainstone	Mud-supported, no imbrication	Tabulate coral, isolated coral, crinoid stem?	Irregular/ scouring bottom contact with LL Facies	1 m in Pinnacle (4)
3	Oval Detritus Facies	F	Grey micrite and greyish white bioclasts	Wackestone	Imbricated, rounded clasts, clasts include 1–1.5 cm oval detritus with minor rounded clasts - averaging 1 cm in diameter	Tabulate coral, fusulinid, isolated coral	Planar lower contact with M	60 cm thick in Pinnacle (2)
4	Brecciated Limestone Facies	Bc	Greyish white and greyish brown	Breccia	No imbrication, polymictic, angular clasts <5 cm in diameter, matrix-supported, clasts include both laminated and structureless limestones	Wood fragment 10 cm long	Planar top contact with Bc Facies	wedge-like, 40 cm thick in Pinnacle (4)
5	Laminated Limestone Facies	LL	Darker grey micrite/ fine materials with white calcite bands. Laminations are dark grey	Mudstone	Lamination, trough stratification	-	Sharp contact and transition with M Facies	Averages 30 cm in thickness

### 4.7 Fossils

**Fig. 5:**
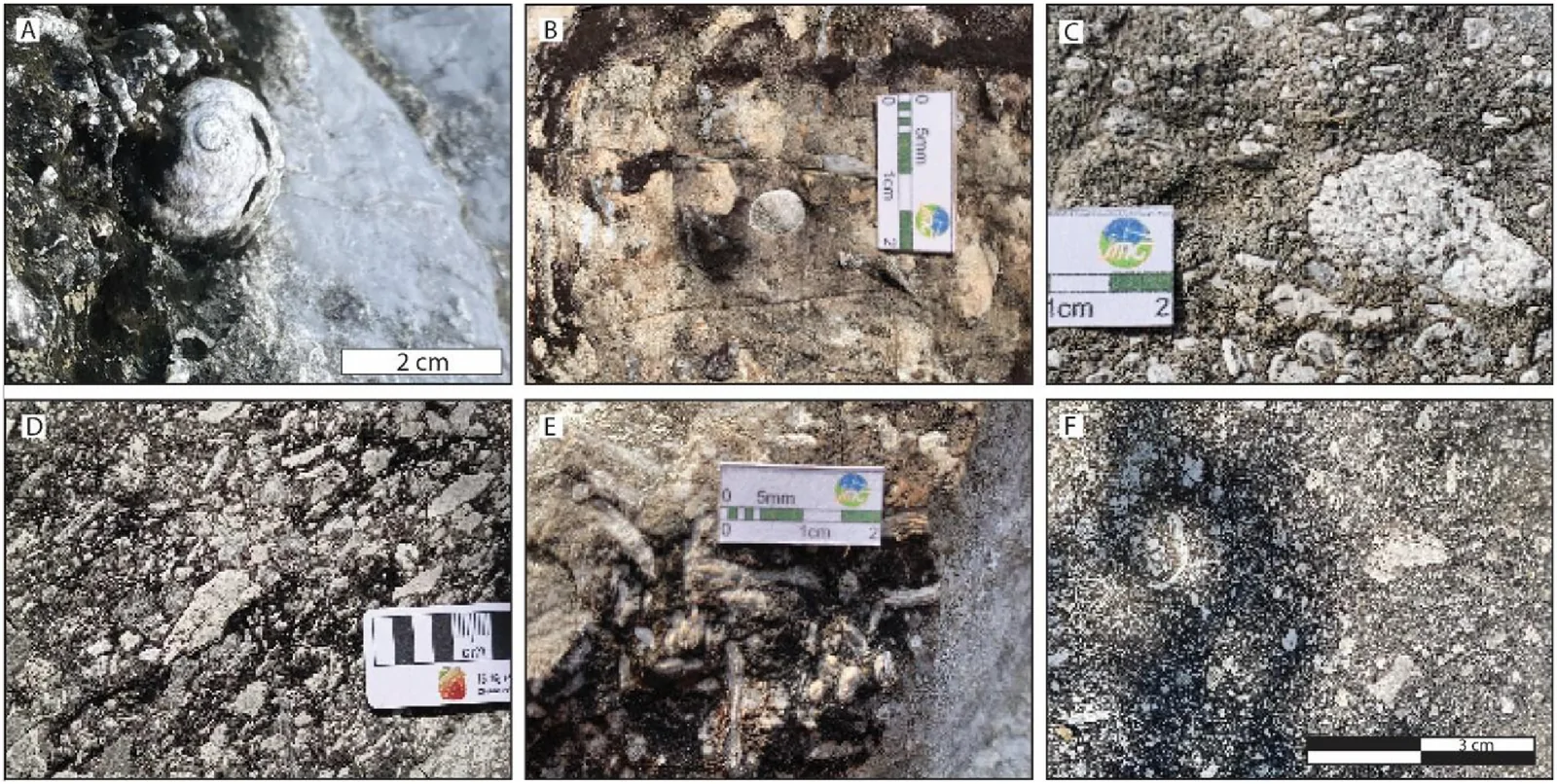
Photographs of fossils identified in this dataset. A) Gastropod, mollusc, B) Isolated horn coral, C & D) Tabulate coral and limestone fragments, E) possibly fusulinids and elongated spicule of isolated coral, and F) brachiopod fragment.

The identified Carboniferous – Permian aged reef fossils are in folder (8). Given the age of the rocks and lithology, the fossils are likely to include fusulinids, corals, brachiopods, crinoids, bryozoans, and algae (Fig. 5).

## Limitations

The limestone in Kinta Valley is highly recrystallized, making thin-section observations challenging. Fusulinids, corals, and other fossils may appear as faint outlines or ghost structures. This recrystallization complicates fossils identification.

## Ethics Statement

This study does not involve experiments on humans, animals or data obtained from social media. All data were acquired though non-invasive geological methods. The authors declare no known competing financial interests or personal relationships that could influence or be perceived to have influenced the findings of this work.

## CRediT Author Statement

**Khor Wei Chung:** Conceptualization, Writing, Investigation, Data Curation; **Mohd Shafeea Leman:** Conceptualization, Formal Analysis, Investigation; **Muhammad Ashahadi Dzulkafli:** Formal Analysis, Funding; **Nurul `Amalina Md. Nor:** Investigation; **Mat Niza Abdul Rahman:** Investigation.

## Data Availability

Mendeley DataCarboniferous – Permian Kinta Limestone Pinnacles, Kampar, Perak, Malaysia (Original data).
